# Supplementary material for: The interactome of intact mitochondria by cross-linking mass spectrometry provides evidence for coexisting respiratory supercomplexes
Source: Mol Cell Proteomics. 2017 Dec 8;17(2):216–32. doi: 10.1074/mcp.RA117.000470 (PMC5795388; doi:10.1074/mcp.RA117.000470)
Supplement: Supplemental Data [file supp_RA117.000470_133922_0_supp_23978_fzffwf.zip › spectra_annotation/mito_spectra_annotation/272-1-3-1-1-1.pdf]

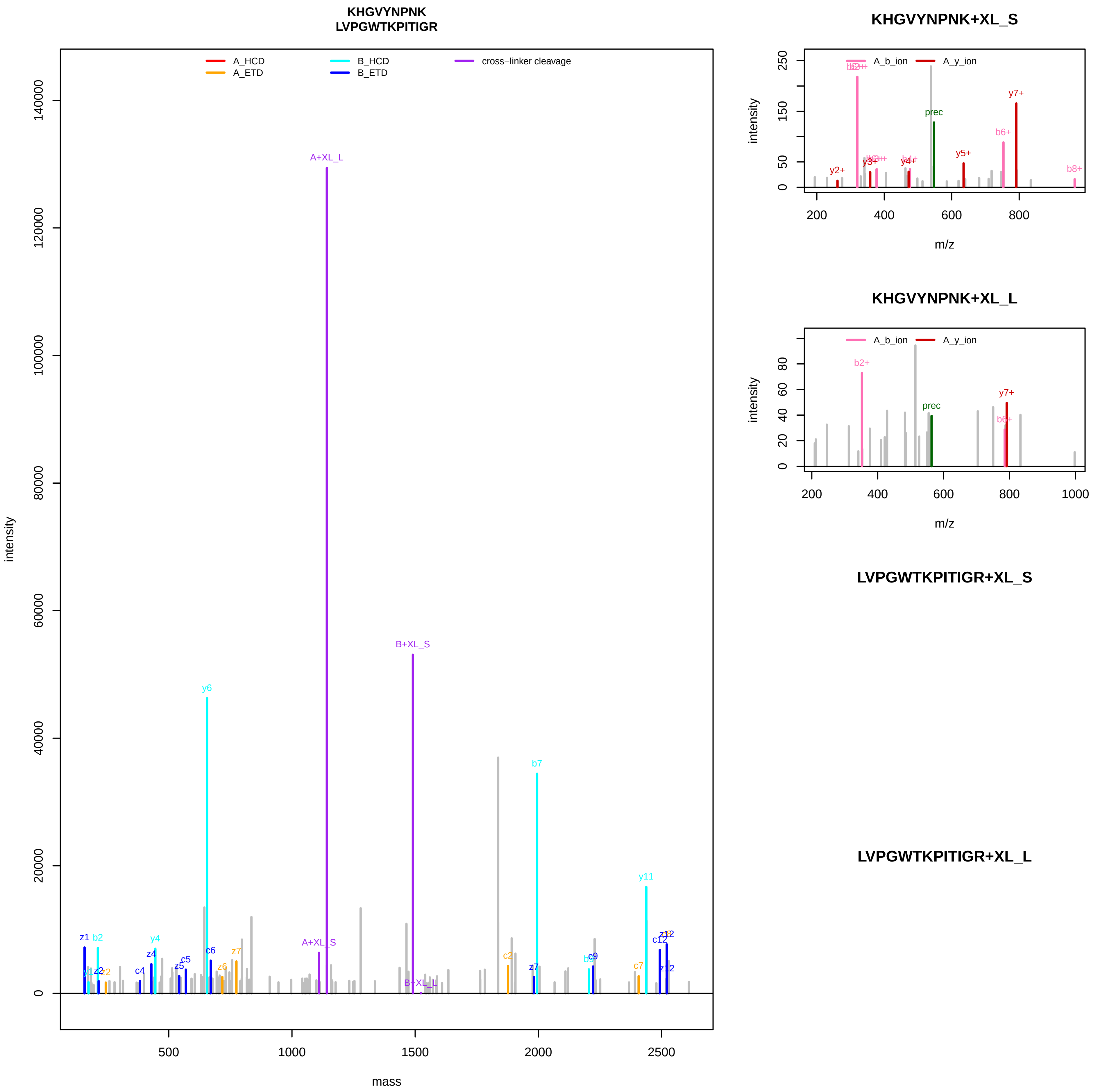

KHGVYNPNK
LVPGWTKPITIGR
A_HCD
A_ETD
B_HCD
B_ETD
cross−linker cleavage
A+XL_L
B+XL_S
A+XL_S
B+XL_L
y6
b7
y11
y4
z1
b2
z2
c4
z4
z5
c5
c6
z6
z7
c2
c7
c9
c12
z12
intensity
mass
0
20000
40000
60000
80000
100000
120000
140000
500
1000
1500
2000
2500
KHGVYNPNK+XL_S
A_b_ion
A_y_ion
prec
y7+
b6+
y5+
y2+
y3+
y4+
b8+
intensity
m/z
0
50
150
250
200
400
600
800
KHGVYNPNK+XL_L
A_b_ion
A_y_ion
b2+
prec
y7+
b6+
intensity
m/z
0
20
40
60
80
200
400
600
800
1000
LVPGWTKPITIGR+XL_S
LVPGWTKPITIGR+XL_L
